# Local Health Departments and the Challenge of Chronic Disease: Lessons From California

**Published:** 2006-12-15

**Authors:** Bob Prentice, George Flores

**Affiliations:** Public Health Institute. Dr Prentice spent nearly two decades at the San Francisco Department of Public Health, including his tenure as director of the Public Health Division. He is currently a senior associate for Public Health Practice at the Public Health Institute and serves as director of the Bay Area Regional Health Inequities Initiative (BARHII); The California Endowment, Los Angeles, Calif. Dr Flores was health officer and public health director in Sonoma and San Diego counties. He served on the Institute of Medicine committees that issued reports on *The Future of the Public's Health in the 21^st^ Century* and *Preventing Childhood Obesity: Health in the Balance.* He is currently a senior program officer with The California Endowment, where he was employed during the research for, and writing of, this article

## Abstract

The essential role local health departments have played in the control of infectious diseases has not been matched with an equivalent contribution in prevention of chronic diseases. Local health departments have attempted to define and build that capacity, but they have been confronted with budget cuts and competing public health priorities, most notably bioterrorism preparedness. This article is based on interviews with local health officials and describes some of the common ways local health departments in California have forged ahead to develop the capacity to engage in comprehensive approaches to chronic disease prevention in spite of the challenges. Additionally, the article highlights future considerations that need to be addressed if these promising trends in chronic disease prevention are to become more widespread.

## Abstract

## Local Health Departments and Chronic Disease

The establishment of local boards of health and health departments throughout the nineteenth and early twentieth centuries created a front line of defense against infectious diseases, which accounted for the preponderance of morbidity and mortality. Today, the nearly 3000 local health departments in the United States have not only continued to play an essential role in infectious disease control but also are increasingly required to address bioterrorism and emerging threats.

It is less clear how the front line of defense applies to chronic disease prevention, where the "vectors" are largely behavior and environment. However, it is a challenge that local health departments must increasingly embrace. Chronic disease accounts for 60% of all deaths worldwide and more than two thirds of all deaths in the United States, nearly half of which occur prematurely (1,2). In California, the Los Angeles County Department of Health Services has documented that chronic disease accounts for 80% of the total burden of disease in Los Angeles County (3), which contributes to lost productivity and high medical care costs. Chronic disease prevention is essential and requires us to examine the changing social and physical environment to better understand the etiologies of chronic illnesses and to determine the most effective prevention strategies. Since chronic diseases often reflect the accumulation of exposures to social and environmental risk factors over the course of generations, they commonly reveal disparities in health status among populations that correspond with patterns of social inequalities.

Local health departments often do not have a well-developed infrastructure to address chronic disease. California health departments are typically organized around categorically funded programs, including vital statistics; maternal, child, adolescent, or family health; environmental health; projects targeting tobacco, nutrition, violence prevention, substance abuse, and injury prevention; and infectious disease control, which commonly includes a public health laboratory as well as separate programs dedicated to tuberculosis, sexually transmitted diseases, HIV/AIDS, other communicable diseases, and epidemiology (4). A survey of local health departments in California during the late 1990s determined that less than 1% of funding and staffing was dedicated to chronic disease prevention (5). In subsequent years, California experienced its largest deficit in history, and budget cuts have cascaded down to local health departments. For example, Los Angeles and San Diego counties, which combined account for more than one third of the state's population (6,7), were forced to dismantle their chronic disease and injury prevention units. Many staff members who worked on chronic disease prevention programs in health departments throughout the state had their efforts shifted to bioterrorism preparedness – the one significantly new source of funding for public health – in order to protect them from program cuts and layoffs.

## The Challenge of Chronic Disease

The absence of a well-developed infrastructure with requisite capacity and resources poses a challenge to local health departments, and this challenge is compounded by the very nature of chronic diseases and the prevention strategies they require. Experience with tobacco control and, more recently, nutrition and physical activity, is instructive; together these two areas account for two thirds of mortality risk factors for chronic disease (8,9).

When tobacco control was equivalent to smoking cessation and education, it fit seamlessly into the clinical and health promotion programs common in local health departments. Tobacco control was most effective, however, when it ventured into environmental change and policy advocacy. Initially, the role of local health departments in those activities was difficult to define. However, when California voters approved a tax on tobacco in 1988, part of the revenues enabled local health departments to expand the scope of their practice to encompass a more complete spectrum of prevention built on smoking cessation and education. These prevention efforts included support for community organizing, formation of anti-tobacco coalitions, development of media campaigns, advocacy for evidence-based legislation to establish smoke-free workplaces and restrict advertising and sales to youth, and support for enforcement of these laws.

Similar ambiguities about local health departments have surfaced more recently as response to the growing epidemic of obesity has progressed beyond nutrition education into strategies employing environmental approaches and policy advocacy to improve nutrition and physical activity. Although there is no current funding source equivalent to the tobacco tax that would support an expanded practice, The California Endowment has funded Healthy Eating, Active Communities, an initiative that involves local health departments, schools, and community coalitions in efforts to improve nutrition and physical activity environments and also includes a statewide policy advocacy component.

## Lessons From California

In spite of substantial challenges posed by chronic disease prevention to local health departments, there are promising trends. Examples of health departments that are creatively building chronic disease prevention infrastructure and programs can be cited based on interviews conducted with senior officials from local health departments in California.

### Examples of promising trends

Although the Los Angeles County health department was forced to dismantle its chronic disease and injury prevention unit because of budget cuts, the unit was revitalized with local funds through the efforts and persistent resolve of senior leadership and a supportive board of supervisors. The newly reconstituted unit brings together program areas that target risk factors — tobacco, physical activity, nutrition, injury, and violence — with special liaisons to cities and communities, schools, and businesses. Reconstruction of the unit includes research and communications support and converges with a strategic planning and leadership development process that prepared staff to engage in the type of collaborative work necessary to address the social and environmental conditions that contribute to chronic disease.

The Shasta County health department in the mostly rural north has for the past 10 years been engaged in a process to reduce the emphasis on clinical services and individual education in order to reinvest in broader population-based approaches. By making creative use of discretionary revenue and using other financing strategies, the department has combined community and regional development efforts with a chronic disease and injury prevention unit; this unit addresses tobacco, nutrition, physical activity, preschool wellness, and injury prevention, and it has a new program on healthy environments that focuses on land use and transportation planning. The Shasta County health department is engaged in a strategic planning process to build the organization around desired outcomes rather than on categorical programs or professions.

The Contra Costa County health department has a well-established commitment to chronic disease prevention. By combining categorical programs with flexible funding, the department has built sustained relationships with communities that encompass multiple issues over time. Examples of such programs include the pioneering Community Wellness & Prevention Program (in existence for more than a decade), Chronic Disease Prevention Organizing Project, and Healthy Neighborhoods Program. Contra Costa County is committed to producing technical assistance tools and publications to assist other local health departments in building their own capacities (10-12).

In its work with neighborhoods in West and East Oakland where the burden of disease and injury is disproportionately high, Alameda County has made community capacity building a centerpiece of its practice. The broad platform enables the health department and community to work together on neighborhood conditions that sustain risk factors for chronic disease and injury, which creates an overarching framework for categorical programs. The health department has also been engaged in a strategic planning process to help staff better understand the social and environmental determinants of health and their relation to health disparities.

The Riverside County health department is part of the rapidly growing Inland Empire and has created a Livable Communities Project that addresses the public health consequences of land use and transportation decisions. Public health evidence and programs are used to underscore the relationship between the built environment and chronic disease.

Two regional approaches are demonstrating how local health departments can work together to build chronic disease capacity. The Bay Area Regional Health Inequities Initiative (BARHII) consists of eight local health departments in the San Francisco Bay Area that have joined together to develop regional strategies to build their internal capacity and to transform practices to better address health inequities. The Central California Regional Obesity Prevention Program (CCROPP) is a collaboration among six local health departments from the Central Valley that are jointly developing strategies focused on obesity prevention.

A common theme in these examples is the importance of local health departments building partnerships. Several local health departments are trying to develop relationships with communities to address social and environmental conditions associated with diseases and risk factors. The growing evidence linking chronic diseases and obesity has generated a surge of public attention, which has reinforced the collaboration between communities and local health departments in addressing social and physical environmental factors that influence nutrition and physical activity. There have been concerted efforts to work with schools on a host of issues ranging from nutrition policies to physical education standards to indoor air quality. There have also been recent significant efforts to revive the public health interest in the built environment, since land use and transportation decisions have major consequences for neighborhood living conditions and health (13).

### Common pathways

Although each local health department is in its own administrative and political environment, there are some common paths these and other health departments have taken to address prevention of chronic diseases.

**Financing**

In the absence of consistent revenues to support comprehensive approaches to chronic disease prevention, health departments have built their capacity by relying on categorical funding supplemented by flexible funding to expand the scope of their work. The most common flexible source is the local general fund, which varies substantially among jurisdictions. Some jurisdictions have made use of state revenue to support core programs, although this approach requires a willingness to disinvest in existing programs.

**Organization**

Organizational strategies have most commonly involved combining separate categorical programs (e.g., tobacco, nutrition, asthma, diabetes, injury prevention, alcohol and drug prevention) into a single unit. This larger unit is then supplemented by a more generalized capacity to work with communities, schools, and other partners.

**Workforce**

Public health nurses, physicians, health educators, dietitians, and other staff are not typically trained or inclined to work on environmental or policy issues. Health departments that have made major commitments to chronic disease prevention have commonly engaged in some form of strategic planning, leadership development, or training to encourage staff to think differently about the nature and scope of their work. Some have assigned staff into dedicated units that over time can influence the larger culture of the health department. New position titles such as Director of Community Capacity Building, Director of Public Health Collaborations, Special Liaison to Schools, or Community Development Coordinator have codified this approach to chronic disease prevention. Recruitment of new employees with skills in community work or land use planning has also been an important complement to the current workforce.

**Leadership**

One factor that seems to have been decisive in the resolve to build chronic disease prevention capacity has been committed local leadership, particularly in the fiscal climate of the past several years. The most effective strategies have been built on a dispersed leadership that has support from the top combined with compatible commitment at the program level.

## Some Considerations for the Future

The likelihood of converting these promising trends seen in some California health departments into a capacity to address chronic disease in a more comprehensive manner will increase if we are able to make progress on several fronts:

- There will need to be reliable statewide revenue to support comprehensive prevention strategies in addition to categorical programs. The current, largely improvisational, developments depend substantially on local funding and support, which disadvantages smaller, rural health departments and jurisdictions where resources for public health tend to be limited. Tobacco taxes are, by design, a diminishing resource, because aggressive measures have helped reduce California smoking rates to 15% (14). Additional taxes on tobacco are a good temporary financing strategy, but they will need to be supplemented by more stable sources in the long run.
- It will be important to have adequate funding and support at the state level so that the development of chronic disease infrastructure does not depend so heavily on local initiative and ingenuity. The two examples of regional approaches suggest that there is value in local health departments learning from one another as they attempt to change their organizations and practice, partnerships that could be amplified considerably with strong state leadership and guidance.
- The training of the current and future workforce will have to include ways in which public health professionals can work with communities and other partners to address the social and environmental determinants of health. New categories of workers — who may or may not originate from schools of public health — will have to be recruited into the public health workforce. Proposed processes to credential the workforce will have to accommodate this expanded pool of skills (15).
- While the Operational Definition of a Functional Local Health Department (16) sanctions the directions described in this paper, accreditation will pose a different set of practical complications as the pressures to develop standards and accountability within reach of most local health departments will conflict with the efforts to expand the boundaries of practice (17). If comprehensive approaches to chronic disease prevention are to become a more generalized capacity within local health departments, they will need to be incorporated into standards and mechanisms for accountability.

## Conclusion

Chronic diseases pose great challenges to public health in the twenty-first century. By collaborating with federal and state agencies, building partnerships with communities and other public and private organizations, and changing social and physical environments, local public health departments can help play a major role in alleviating the burden of chronic disease in the coming years.
